# Sports Obstetrics: Implications of Pregnancy in Elite Sportswomen, a Narrative Review

**DOI:** 10.3390/jcm11174977

**Published:** 2022-08-25

**Authors:** Ariadne L’Heveder, Maxine Chan, Anita Mitra, Lorraine Kasaven, Srdjan Saso, Tomas Prior, Noel Pollock, Michael Dooley, Karen Joash, Benjamin P. Jones

**Affiliations:** 1 Department of Surgery & Cancer, Imperial College London, London W12 0NN, UK; 2 Queen Charlotte’s and Chelsea Hospital, Imperial College NHS Trust, Du Cane Road, London W12 0HS, UK; 3 Institute of Sport, Exercise & Health, University College London, London W1T 7HA, UK; 4 British Athletics, National Performance Institute, Loughborough, LE11 3TU, UK; 5 The Poundbury Clinic, Dorchester, Dorset DT1 3FD, UK

**Keywords:** athlete, exercise, olympics, obstetrics, antenatal, intrapartum, postpartum, guidance

## Abstract

Increasing numbers of females are participating in elite sports, with a record number having competed at the Tokyo Olympic Games. Importantly, the ages of peak performance and fertility are very likely to coincide; as such, it is inevitable that pregnancy will occur during training and competition. Whilst there is considerable evidence to promote regular exercise in pregnancy, with benefits including a reduction in hypertensive disorders, gestational diabetes, and reduced rates of post-natal depression, few studies have been conducted which include elite athletes. Indeed, there are concerns that high-intensity exercise may lead to increased rates of miscarriage and preterm labour, amongst other pregnancy-related complications. There is minimal guidance on the obstetric management of athletes, and consequently, healthcare professionals frequently adopt a very conservative approach to managing such people. This narrative review summarises the evidence on the antenatal, intrapartum, and postpartum outcomes in elite athletes and provides recommendations for healthcare providers, demonstrating that generally, pregnant athletes can continue their training, with a few notable exceptions. It also summarises the physiological changes that occur in pregnancy and reviews the literature base regarding how these changes may impact performance, with benefits arising from pregnancy-associated cardiovascular adaptations at earlier gestations but later changes causing an increased risk of injury and fatigue.

## 1. Introduction

Moderate physical activity during pregnancy is beneficial and should be encouraged [1,2,3]. Whilst a number of guidelines recommend regular exercise in pregnancy, recommendations for elite athletes remain limited [1,3,4,5,6,7]. The proportion of female participants in the Olympic Games has consistently risen, to a high of 49% (*n* = 5386) in 2021 compared with 23% (*n* = 1566) in 1984 [8,9]. With increasing numbers of female athletes competing throughout their twenties and thirties [10], where the chronologies of optimal performance and peak fertility coincide, pregnancy is very likely to occur during periods of training and competition [11]. Athletes may defer pregnancy until after their prime performance potential declines, often when their reproductive capacity has simultaneously diminished, and obstetric risks have increased, or plan pregnancies between competitions, requiring consideration of the impact on their intense training schedules, before, during, and immediately following pregnancy [12].

A range of maternal physiological adaptations occurs during pregnancy. These, and their implications on performance, are summarised in Table 1. Whilst the International Olympic Committee (IOC) has produced a consensus statement on exercise during pregnancy and postpartum [13], there is little guidance regarding the obstetric management of athletes [7]. Despite the theoretical risks associated with high-intensity exercise during pregnancy, such as fetal growth restriction (FGR) and miscarriage [14,15], there is uncertainty about the optimum management of the pregnant athlete [16]. Consequently, healthcare professionals are often cautious and may advise against high-intensity exercise in pregnancy [17]. As such, this narrative review aims to evaluate the impact of high-intensity exercise during pregnancy and postpartum [18] and provide obstetric guidance for managing elite athletes. Given the limited good-quality evidence specific to elite athletes [7,19], evidence from studies looking at varying intensity levels has been considered. The MeSH terms; sport*, athlete, exercise, pregnan*, miscarriage, preterm birth, incontinence, pelvic floor, antenatal, postnatal and physiological changes were searched for in the following databases: MEDLINE, Google Scholar, Cochrane Database of Systematic Reviews.

## 2. First Trimester

Following conception, there is a multitude of implications requiring consideration for elite athletes. A concern regarding high-intensity exercise during the first trimester is the potential impact of increased body temperature during organogenesis, given evidence that maternal hyperthermia above 39 °C may be teratogenic [28,29]. Not only does moderate-intensity exercise increase average core body temperature by 1.5 °C after 30 min [23], but pregnancy itself raises basal metabolic rate [14], the combination of which exposes a greater risk of hyperthermia [16]. A systematic review reported that maternal hyperthermia in the first trimester increased the risk of neural tube defects (OR 1.92; 1.61–2.29) [30]; however, the most prevalent aetiology of hyperthermia in the included studies was febrile illness. There was no evidence of the detrimental effects of exercise on fetal development. No association was demonstrated between moderate exercise (yoga or aerobic exercise performed 1–7 days per week, for 15–50 min per session at a low-to-moderate intensity) and hyperthermia (increase by ≥2 °C) (15 studies, *n* = 447) or congenital anomalies (14 studies, *n* = 78,948) [31]. The evidence was, however, of ‘low’ or ‘very low’ quality, and several of the papers included in the meta-analysis began the exercise intervention in the second and third trimesters when effects on organ formation are less likely. Additionally, none of the studies included in this paper involved elite athletes nor women exercising to a comparable level. A systematic review including 12 studies (exercise interventions: running and aquatic aerobics, duration 15–45 min, 51–90% maximum heart rate (HRmax)) found the highest single and mean core temperatures to be 38.9 °C and 38.3 °C, which are below the teratogenic threshold [32]. The authors conclude that pregnant women can safely engage in exercise for up to 35 min on land at 80%–90% HRmax at 25 °C and 45% relative humidity, or up to 45 min in water (≤33.4 °C). Safe core temperature levels with moderate-intensity exercise for 45 min at 32 °C have also been demonstrated [33]. Crucially, it remains unknown at which intensity and duration of exercise and what environmental temperature and humidity the upper limit for maternal core temperature is exceeded, and whether elite athletes could reach this threshold. Until such evidence is available in the literature, caution should be exercised to keep core temperature < 39 °C.

### Miscarriage

There is conflicting evidence regarding high-intensity exercise and miscarriage risk [6,7,15]. One study assessing outcomes following in vitro fertilisation concluded that women who exercised over four hours per week had double the risk of miscarriage [34]. Furthermore, Madsen et al. assessed prenatal exercise habits in over 90,000 women before and after having a miscarriage and found a higher risk of miscarriage with increasing exercise volume; with a hazard ratio of 3.7 (95% CI 2.9–4.7) for those exercising over seven hours per week [35]. However, this association was not significant in the secondary analysis, which only included those interviewed prospectively [36]. Moreover, no difference in miscarriage rates was found when comparing 34 international Norwegian athletes with active controls who undertook >150 min of exercise per week (3% vs. 8%, *p* = 0.18) [37]. In addition, a recent systematic review and meta-analysis found that, compared with no exercise, prenatal exercise (the frequency of which ranged from 1–7 days per week, from low to vigorous intensity, with a duration from 10 to 95 min per session) was not associated with increased miscarriage risk (OR 0.88, 95% CI 0.63 to 1.21, I^2^ = 0%), nor was there a relationship identified between the risk of miscarriage and frequency, duration, intensity, or volume of exercise [38]. Furthermore, a meta-analysis specifically focusing on elite athletes did not find increased rates of miscarriage (*n* = 57, OR 0.32, 95% CI 0.07–1.34) [7]. Notably, the evidence was of “very low” quality due to the risk of bias and inconsistency. Nevertheless, there is no scientific reason why exercise would increase the risk of pregnancy loss; indeed, Meah and colleagues argue that recurrent miscarriage should not be considered a contraindication to engaging in physical activity [39].

## 3. Second and Third Trimesters

### 3.1. Fetal Growth and Birth Weight

It has been proposed that female athletes are at risk of fetal growth restriction (FGR), primarily due to the physiological response to regular strenuous exercise, which causes repeated, albeit temporary, redistribution of blood flow away from the uterus toward skeletal muscles [15]. Such women are also at risk of insufficient gestational-weight-gain (GWG), which could indirectly predispose them to FGR [16]. However, a systematic analysis in elite female athletes found ‘very low’ certainty evidence indicating an increased odds of excessive prenatal weight gain versus active/sedentary controls (*n* = 1763; odds ratio, 2.47; 95% confidence interval, 1.26–4.85; I^2^ = 0%) [7]. Whilst there is evidence of intense exercise resulting in FGR in animal studies [16,17,18,19,20,21,22,23], data in humans remain inconclusive [40]. One prospective international study (*n* = 3513) found that daily vigorous exercise (leading to heavy breathing or being out of breath) was an independent risk factor for small-for-gestational-age (SGA) on customized growth charts, with an adjusted OR of 3.3 (CI 1.5–7.2) [41]. Conversely two retrospective studies comparing elite athletes (*n* = 34 and *n* = 40) to controls found no significant difference in birthweights [37,42]. A further meta-analysis focusing specifically on the effect of vigorous activity (defined as being at least 70% of HRmax or an activity in which a conversation cannot be maintained) in the third trimester on birth weight, including 8 cohort studies (*n* = 7225) and 5 RCTS (*n* = 623), found no significant difference in birth weight for babies of mothers who engaged in vigorous physical activity and those who did not (mean difference 8.06 g, 95% CI 57.44 to 73.55, *p*  =  0.79, I^2^  =  53.92) [43]. Similarly, no association between preconception elite competitive sporting exposure and birth weight (*n* = 2145, four studies; MD, 20.6; 95% CI −76.1–117.2; I^2^ =13%) was found in a very recent meta-analysis [7].

Despite the convincing evidence that exercising to an elite level pre-conception and during pregnancy does not lead to SGA, the Royal College of Obstetricians and Gynaecologists identifies ‘daily vigorous exercise’ (>5 h/week, moderate intensity) as a major risk factor for SGA, advising serial ultrasound assessment of fetal growth [44]. Canadian guidance states exercise is not associated with FGR but highlights that this does not include studies where women are exercising at levels significantly above recommendations [3]. Evidence from a recent meta-analysis in elite athletes, whilst of “very low” quality, is reassuring and suggests this guidance may need to be reviewed [7]. Nevertheless, given the lack of good quality evidence in those exercising at high intensity, it may be reasonable to consider starting aspirin in the first trimester if there are concerns regarding placental insufficiency and perform serial growth scans if exercising over five hours per week in order to identify SGA [44]. This is due to concerns that vigorous or high-intensity exercise could have negative impacts on fetal well-being in a pregnancy complicated with uteroplacental insufficiency due to reduced blood flow to the fetoplacental unit and fetal hypoxia [39].

### 3.2. Fetal Heart Rate, Umbilical and Uterine Blood Flood

Evidence has suggested that high-intensity exercise may cause alterations in fetal heart rate (FHR), although the clinical significance of this remains uncertain as no subsequent adverse outcomes have been observed [20,26]. A metanalysis including studies with mixed exercise levels providing very low-quality evidence demonstrated a mean increase in FHR of 6.35 beats per minute (bpm) during and 4.05 bpm following acute exercise (19 studies, *n*= 348 women, 95% CI 2.30 to 10.41, I^2^ = 95%, *p* = 0.002) and (65 studies, *n* = 1845 women, 95% CI 2.98 to 5.12, I^2^ = 83%, *p* < 0.00001), respectively [26]. A meta-regression analysis identified a curvilinear dose-response relationship between the volume of exercise (MET minutes per session) and FHR both during and following exercise up to a certain point (peak FHR 9.8 bpm above baseline at 138.3 MET minutes and 7.2 bpm at 174.6 MET minutes, respectively) after which FHR plateaued or increased to a lesser extent [26]. A case series of six Olympic athletes performing submaximal workloads between 23 and 29 weeks gestation demonstrated that fetal bradycardia occurred when the mean uterine artery blood flow was <50% of the initial value or when women reached ≥90% HRMax, but not below this level, which resolved within 10 min of activity cessation [12]. One woman developed haemolysis–elevated liver enzymes–low platelets syndrome at 35 weeks and was induced; otherwise, there were no other adverse outcomes. Indeed, in the aforementioned meta-analysis, there was “very low” quality evidence from seven studies (*n* = 755 women) that fetal bradycardia occurred during 1% of exercise sessions (95% CI 0.01–0.03, I^2^ = 76%); notably, these events lasted <2 min and were not considered to be clinically relevant. Additionally, women exercising in the supine position provided 17% of the cases of reported bradycardia during exercise, suggesting this position should be avoided [26].

The same meta-analysis provided further evidence that exercise is not detrimental, demonstrating no changes in umbilical artery pulsatility index (PI) and other measures of uterine blood flow following acute exercise sessions; however, only one of the 91 studies evaluated included exercise at >90% maximal intensity and demonstrated a 12% decline in uterine artery systolic: diastolic ratio following exercise [26]. Rafla and colleagues investigated the effect of exercise on placental blood flow and FHR, both in healthy subjects and women with FGR [45,46]. The study utilised exercise stress tests on bicycle ergometers, with increasing workload each minute until the patient achieved 70% of her submaximal exercise. The studies showed that the mean PI value after 30 min of recovery was significantly higher than the mean baseline rest value in cases of FGR but not in healthy patients in whom, after an initial decrease in resistance, blood flow returned to normal within 20 min of recovery time [45,46]. Furthermore, in high-risk pregnancy, there were 10 cases of bradycardia following exercise, but none in the low-risk group [45]. As there may be risks associated with vigorous activity in high-risk pregnancies, particularly those with FGR, this reaffirms the recommendation to undertake fetal growth scans throughout athletes’ pregnancies. Additionally, the long-term effects of exercising ≥90% HRMax, given the impact on uterine artery blood flow and FHR, albeit transient, need to be further investigated; Beetham and colleagues recommend avoiding exercising at a perceived exertion relative to ≥90% HRMax until further studies confirm its safety [43].

### 3.3. Pre-Eclampsia and Gestational Hypertension

Exercise has been shown to have a beneficial impact on hypertensive disorders in the general obstetric population [2,3,6]. A meta-analysis including 17 RCTs found women randomised to 30–60 min of aerobic exercise, two-seven times per week until >35 weeks gestation, had a significantly lower incidence of pregnancy-induced hypertension or pre-eclampsia (5.9% vs. 8.5%; RR = 0.70, 95% CI 0.53–0.83, *n* = 2517) [47]. However, concerns have been raised with high-intensity exercise, following evidence from a large prospective cohort study (*n* = 85,139) which identified that women who exercised >270 min/week in the first trimester were at higher risk of severe pre-eclampsia compared to their non-exercising counterparts (adjusted OR 1.65 (95% CI: 1.11–2.43) [48]. This effect was only specifically seen in severe pre-eclampsia, with no overall association between physical activity and pre-eclampsia. Additionally, this effect has not been replicated by other studies [49]. Contrastingly, a meta-analysis suggests an inverse association between high levels of physical activity and severe pre-eclampsia risk, with a 20–35% and 20% relative risk reduction for women with the highest physical activity level pre-pregnancy and during early pregnancy, respectively [50]. Regarding pre-pregnancy exercise, a non-linear association between physical activity and pre-eclampsia was seen, with a 40% reduction in risk up to five-six hours/week but no further risk reduction at activity levels beyond this [47]. Whilst exercising at an elite level is more likely to be preventative than causative of pre-eclampsia, should severe pre-eclampsia develop with severe features such as FGR, women should avoid moderate to vigorous physical activity given previous, albeit dated, evidence of reduced placental blood-flow in these cases [39,51,52].

### 3.4. Placental Abruption

One multicentre case-crossover study (*n* = 663 women from seven hospitals) interviewed women who had had a placental abruption regarding their exercise habits both one hour and one week prior to symptom onset [53]. The instantaneous incidence rate ratio of placental abruption within an hour of moderate (dancing) or heavy (sprinting) physical exertion was lower for those who habitually engaged in moderate or heavy physical activity over three times per week in the year before pregnancy (rate ratio (RR) = 3.0, 95% CI: 1.6, 5.9) compared with more sedentary women (RR = 17.3, 95% CI: 11.3, 26.7; P for homogeneity < 0.001). Whilst exercise may be protective against abruption, the same study found that the risk of placental abruption was 7.8 (95% CI: 5.5, 11.0) times greater in the hour following moderate or heavy physical exertion, with the highest risk being after heavy-intensity exertion (rate ratio = 13.7, 95% CI: 7.0, 26.5). Notably, however, only 54% of the participants engaged in any moderate to vigorous activity in the week before the placental abruption; thus, it is not clear whether avoiding exercise would only delay, rather than prevent, the inevitable outcome of placental abruption. The avoidance of vigorous activity is not advised to prevent abruption [39].

## 4. Intrapartum Considerations

### 4.1. Preterm Birth

Antenatal exercise does not appear to increase the risk of preterm birth (PTB) in the general population [54] and may well be protective against PTB [4]. The IOC evaluated data from six randomized studies from 2009 to 2014 and found no difference in rates of PTB between those engaging in aerobic exercise and controls [15]. Regarding women exercising at an elite level, a meta-analysis found that aerobic exercise, up to 90 min, four times a week did not increase PTB risk [55]. Similarly, a retrospective case-control study of 40 elite athletes found no difference in PTB rate compared to controls [42], and neither did a study evaluating 30 Finnish endurance athletes whose pregnancy outcomes were compared with non-athletic nulliparous women [56]. Some studies have additionally demonstrated that physical activity exerts a prophylactic effect on delivering preterm [57]. Beetham et al. conducted a meta-analysis specifically focusing on the effects of vigorous activity in the third trimester finding a small but significant increase in gestational age at delivery (difference = 0.21 weeks; 95% CI 0.15 to 0.27, *p* < 0.001, *n* = 4281) and reduced risk of prematurity in the babies of mothers who engaged in vigorous physical activity (RR  = −0.20; 95% CI −0.36 to −0.03, *p*  =  0.03, respectively) [43].

Anecdotal concerns regarding increased risk of PTB in women who weight-lift and partake in cross-fit, which are increasingly popular activities, require consideration. Although no data have been collected from elite performers, a large study using the Danish National Birth Cohort (*n* = 62,803) found a dose–response relationship between self-reported total daily burden lifted and PTB with an OR of 1.50 (95% CI 1.03 to 2.19) with loads over 1000 kg/day [58]. This finding is further supported in a recent meta-analysis where women standing for >3 h per day had a 10% increase in the odds of PTB [59]. Further work is required related to occupational activity and whether there are relevant psychosocial factors involved that are similar to elite athletes for whom physical activity is also their occupation. Thus far, weight training for exercise purposes has not been found to be associated with any adverse pregnancy outcomes.

### 4.2. Labour and Mode of Delivery

It has been suggested that exercise may decrease or not impact labour duration [15]. Amongst healthy pregnant women, regular physical activity has been shown to slightly increase likelihood of vaginal delivery (RR = 1.12, 95% CI 1.01–1.24; *p* = 0.041) and reduce the risk of caesarean section (CS) (RR = 0.66, 95% CI 0.46–0.96; *p* = 0.028) [60]. This is supported by two meta-analyses: one finding that women randomised to 30–60 min of aerobic exercise two-seven times/week until at least 35 weeks had a significantly lower rate of delivery by CS compared with controls (RR = 0.84, 95% CI 0.73–0.98) [47]. The other demonstrated that antenatal exercise decreased the probability of having an instrumental delivery by 24% (OR = 0.76, 95% CI 0.63 to 0.92) [61]. No other significant associations were seen between exercise and length of labour, induction of labour, CS, vaginal tears, injury, or musculoskeletal trauma [61].

Specifically concerning athletes, it has been suggested that stronger abdominal muscles may enable more effective pushing, thereby reducing the duration of the second stage of labour [15]. Conversely, high-intensity exercise has been shown to cause hypertrophy of pelvic floor muscles, which could hypothetically increase the risk of obstructed labour [62]. However, a comparison between 40 elite athletes and a control group did not identify any difference in the mode of delivery (MOD) [42]. Two other studies in which outcomes were compared between nulliparous athletes and non-athletes found no difference in the length of phases of labour nor in CS rate [56,63]. Whilst there was no difference in perineal trauma when all athletes were compared to controls, there was a significantly higher incidence of 3^rd^- and 4^th^-degree tears in low-impact athletes compared to high-impact athletes (23.7% vs. 5.1%, *p* = 0.01) [63]. A recent meta-analysis found very low certainty evidence of no association between preconception competitive sporting and prolonged first stage of labour (*n* = 398), prolonged second stage (*n* = 248), caesarean sections (*n* = 324), instrumental deliveries (*n* = 77), episiotomy (*n* = 163), and perineal tears (second-to-fourth degree) (*n* = 411) in elite athletes [7].

## 5. Postnatal Implications

Whereas many pregnancy-associated physiological changes persist for four-six weeks following delivery [14,23], the most profound physiological changes occur soon after delivery [20]. Traditionally women have not been encouraged to exercise in the postpartum period, but the IOC recognise this is an arbitrary time point, and many elite athletes begin exercising within 6 weeks [64].

### 5.1. Pelvic Floor Dysfunction and Incontinence

Around 15–30% of women experience urinary incontinence (UI) in the first year postpartum, irrespective of MOD [65]. Female athletes have a theoretically increased risk of UI, anal incontinence (AI), and pelvic organ prolapse because of large increases in intra-abdominal pressure during strength training, which causes additional strain on pelvic floor musculature [13,66,67]. However, a study on elite athletes (*n* = 40) did not find any significant difference in AI or UI prevalence compared to controls, both during pregnancy and at 6 weeks postpartum [42]. Neither did two recent metanalyses specifically focusing on elite athletes [7,19], nor a recent retrospective study comparing international athletes (*n* = 34) with active controls, with rates of UI in the postpartum period of 21% and 27%, respectively (*p* = 0.78) [37]. Another important consideration is the risk of pelvic organ prolapse, particularly in those participating in high-impact sports such as Cross-Fit, weightlifting, and running, especially as prolapse symptoms can be a deterrent to returning to training postpartum [68,69]. Whilst there is limited evidence on the impact of pregnancy on pelvic floor dysfunction and UI, given the increased prevalence of UI in athletes out-with pregnancy, most notably in higher impact sports [66,67,68,69], they remain a high-risk group, and as such warrant pro-active screening and pelvic floor exercise advice from a women’s health physiotherapist.

### 5.2. Breastfeeding

Regular exercise does not appear to adversely impact breast milk volume, composition, or infant weight [2,64,70], with some research suggesting it may increase milk volume and energy content [71]. A study assessing the impact of running on breastfeeding in competitive runners found that 84.1% exercised whilst breastfeeding, compared to only 30.9% who exercised in the third trimester [72]. Overall, 84.4% of runners reported that running had no effect on breastfeeding, and 7.8% reported a positive relationship. More recently, a meta-analysis focusing on health outcomes after pregnancy in elite athletes found very low certainty evidence of no association between pre-pregnancy elite athletic exposure and initiation of breastfeeding postpartum, nor the length of breastfeeding postpartum [19]. Importantly breastfeeding has been shown to have a deleterious effect on bone mineral density (BMD) due to the loss of calcium from the maternal bone mass [73,74], which may increase the risk of bone stress injury [37,75,76]. Whilst the link between breastfeeding and injury is weak, with no evidence of this association among elite athletes, breastfeeding athletes should take precautions to increase impact load gradually, with appropriate muscle strengthening to support a return to impact activities.

### 5.3. Injury

Several studies have identified a potential propensity for injury in elite athletes in the postpartum period, in particular, a risk for stress fractures [19,37,42,72,76]. This may relate to a reduction in BMD due to calcium loss through breastfeeding, but there are other potential considerations [19]. Bone stress injury is common in elite endurance female athletes [77], and an increase in training intensity after any period of reduced training load may be associated with increased injury risk. Increased joint laxity has also been identified in postpartum females, which could potentially contribute to general injury risk [19,27]. In their recent metanalysis on elite athletes, Kimber et al. recognised the rate and nature of injuries amongst postpartum athletes to be of concern; with very low-quality evidence from three studies (*n* = 179) [37,72,76] that 16 injuries were sustained among 14 athletes, with a disproportionately high rate of sacral fractures compared to the non-postpartum population which was felt to be related to breastfeeding and engagement in high impact sports [19]. Until further conclusive research is carried out, it would be sensible to risk assess for injury in the postpartum period with a trained women’s health physiotherapist.

### 5.4. Postnatal Depression

Evidence remains conflicting regarding the prevalence of PND amongst elite sportswomen [64]. Whilst some studies show no benefit to PND following exercise intervention, including an RCT assessing the impact of exercise sessions three times/week [78], others, which include meta-analyses, have demonstrated physical activity to be protective [79,80,81,82]. This effect has been identified in elite athletes, where female competitive runners had a lower prevalence of PND when they ran during breastfeeding compared to those athletes who did not (6.7% vs. 23.5%, *p* = 0.051) [72]. As stress may exacerbate PND, it is important to acknowledge that some athletes may feel anxiety regarding returning to a competitive level following childbirth [83]. Conversely, not competing or training or pressure to refrain from such activity may also have psychological sequalae, highlighting the need for individualisation depending on interindividual motivations and perceptions.

## 6. Neonatal Outcomes

Exercising antenatally or postpartum has not been identified to adversely impact neonatal outcomes. A meta-analysis of 13 low-quality RCTs (*n* = 6387) did not show an association between antenatal exercise and perinatal mortality (OR 0.86, 95% CI 0.49 to 1.52, I^2^ = 0%) [38]. Encouragingly, a systematic review and meta-analysis including 135 studies with varied exercise regimens found antenatal exercise had no effect on gestational age, birth weight, neonatal hypoglycaemia, cord gases, hyperbilirubinaemia, Apgar scores, neonatal body composition, childhood obesity, or developmental milestones [54]. Similarly, a multicentre RCT demonstrated that regular moderate-intensity exercise during pregnancy did not adversely impact neurodevelopment at seven years of age [84]. Whilst there are limited data on elite athletes, the aforementioned Norwegian study comparing elite athletes with controls found no difference in the birthweight or APGAR scores between babies born to athletes (*n* = 29) versus controls (*n* = 29) [37].

## 7. Impact of Pregnancy on Performance

Whereas the impact of exercise on pregnancy remains the main focus for healthcare professionals, the impact of pregnancy on performance is seldom addressed. Examples of sportswomen competing during pregnancy and postpartum include Serena Williams, who won the Australian Open whilst pregnant, and Paula Radcliffe and Jessica Ennis-Hill, whom both won elite events 10 and 13 months after childbirth, respectively [5]. Although there is little evidence amongst elite athletes, several studies demonstrate that women can maintain high levels of performance during pregnancy and return to pre-pregnancy fitness levels post-delivery [10,21,37,76]. It has even been postulated that some pregnancy-induced physiological changes may enhance exercise potential [56].

Maximum oxygen consumption (VO2max) is closely related to cardiorespiratory fitness and is a key determinant of elite performance in endurance sports [85]. It can be defined as the maximum integrated capacity of the pulmonary, cardiovascular, and muscular systems to uptake, transport and utilize oxygen, respectively [86]. Endurance athletes have a high VO2 max, which enables high rates of blood flow and oxygen transport to muscles [87]. Whether the cardiorespiratory changes that occur during pregnancy affect VO2max is undetermined. Whilst it has been argued that pregnancy-associated cardiorespiratory changes, in particular increased cardiac output (CO), could improve performance [20,21,88], a clinical review concluded there was no significant impact upon exercise [11]. The increase in CO is most pronounced between 20–28 weeks before it declines towards term as stroke volume decreases [21]. This physiology likely underpins the findings of a study that identified a significant reduction in VO2max in the third trimester compared to the first and second trimesters [89].

Whilst not specifically assessing VO2 max, other studies, such as one that included 41 pregnant athletes training at moderate–high intensity [10] and another that assessed the world’s most successful cross-country skier’s training volumes, demonstrated that high levels of fitness and intense training could be maintained throughout pregnancy; with training volumes of 89% and 49% of pre-pregnancy values in the second and third trimesters, respectively [76]. This is reaffirmed by data from 30 Finnish endurance athletes, where 23 continued to train, and 18 competed up to a mean of 23 weeks and 8 weeks gestation (range 7–39 and 0–25 weeks), respectively [56]. Sixteen athletes did not perceive any difference in performance, whereas three and seven felt it improved and worsened performance, respectively.

Postpartum, a study that compared non-pregnant and pregnant athletes (*n* = 40) who maintained moderate-to-high intensity exercise throughout pregnancy identified a significant increase in VO2 max at 12–20 weeks postpartum, which was maintained 36–40 weeks following delivery [88]. Contrarily, a recent study in recreational exercisers found decreases in both VO2max and leg strength from pre-pregnancy to six-weeks postpartum, most of which recovered by 27 weeks postpartum [90]. The IOC concluded that after pregnancy, VO2max and strength are reduced at 6 weeks postpartum compared to pre-pregnancy [64].

Regarding fitness levels postpartum, a retrospective study on athletes showed that 77% continued to compete at the same level following childbirth [42]. In the aforementioned Finnish study, 18 women continued to compete for 8.2 months (2–24 months) following delivery. Two had improved exercise performance, 11 maintained the same level, and five were unable to achieve the same pre-conception level [56]. A recent study comparing international athletes (*n* = 34) with active controls identified that 44% reported their performance level remained static postpartum compared with pre-pregnancy, whereas 15% and 26% reported improved and worsened performance, respectively [37]. A recent meta-analysis found “very low” certainty evidence from five studies that athletes participating in elite spot pre-pregnancy may experience improved performance in the postpartum period [19].

Several other pregnancy-associated physiological changes may impact athletic performance [14,23]. As summarised in Table 1, concerns include the impact of anaemia on endurance [11,13,23], changes in the centre of gravity, and increased joint laxity, which may limit performance, and as previously discussed, injury risk [19,27].

Exercise impacts iron absorption, metabolism, and regulation, predominantly through its impact on the key iron regulatory hormone, hepcidin [91]. Additionally, endurance training increases blood volume by as much as 35% [11,89]. As such, it is unsurprising that the prevalence of iron deficiency in female athletes is between 15 and 35% [92]. Exercising during pregnancy, therefore, likely exacerbates the pre-existing physiological anaemia associated with pregnancy and may impact elite athletes’ ability to partake in high-intensity training compared to recreational exercisers [11]. Pivarnik et al. compared blood volumes and haematology profiles between nine aerobically trained women who exercised throughout pregnancy, and five healthy sedentary women at 25 weeks, 36 weeks, and 12 weeks postpartum [92]. They found significantly increased blood volumes in the active group (*p* < 0.01) at all time points. Red cell volume, haemoglobin, and total red blood cell counts also increased with blood volume, yet these levels were significantly reduced during pregnancy compared to 12 weeks postpartum. However, an RCT that compared haematological variables in 80 sedentary women undertaking light exercise 35–40 min three times/week with 80 who undertook no exercise found no difference in the incidence of anaemia [24]. Incidentally, all women received iron supplementation. This is significant as a recent meta-analysis concluded supplemental iron improves iron status and aerobic capacity of iron deficient non-anaemic endurance athletes [93].

GWG may also impede the ability to maintain optimum exercise intensity [11]. This was demonstrated when comparing the physiological responses between non-weight-bearing and weight-bearing exercise at 34 weeks gestation and 7–8 weeks postpartum (*n* = 10). Whereas there was a reduction in treadmill performance, there was no change in VO2max observed during non-weight bearing cycle ergometry, suggesting GWG is responsible [94]. Larew et al. evaluated exercise attainment and weight gain over 12 months in 83 pre-menopausal women and found lower rates of weight gain to be significantly associated with longer treadmill endurance times (r =−0.21, *p* = 0.04) [95]. As such, it is likely that GWG may have a deleterious effect on exercise performance.

There are other sociological considerations, such as access to childcare, change in the daily routine, loss of financial earnings through missed competition, or a lack of sleep in the postpartum period, which can impact the ability to train and recover. Health professionals and coaches supporting elite female athletes should be cognisant of these factors that will impact training and potentially performance.

## 8. Assessment and Management of the Elite Athlete during Pregnancy and Postpartum

**Figure 1:**
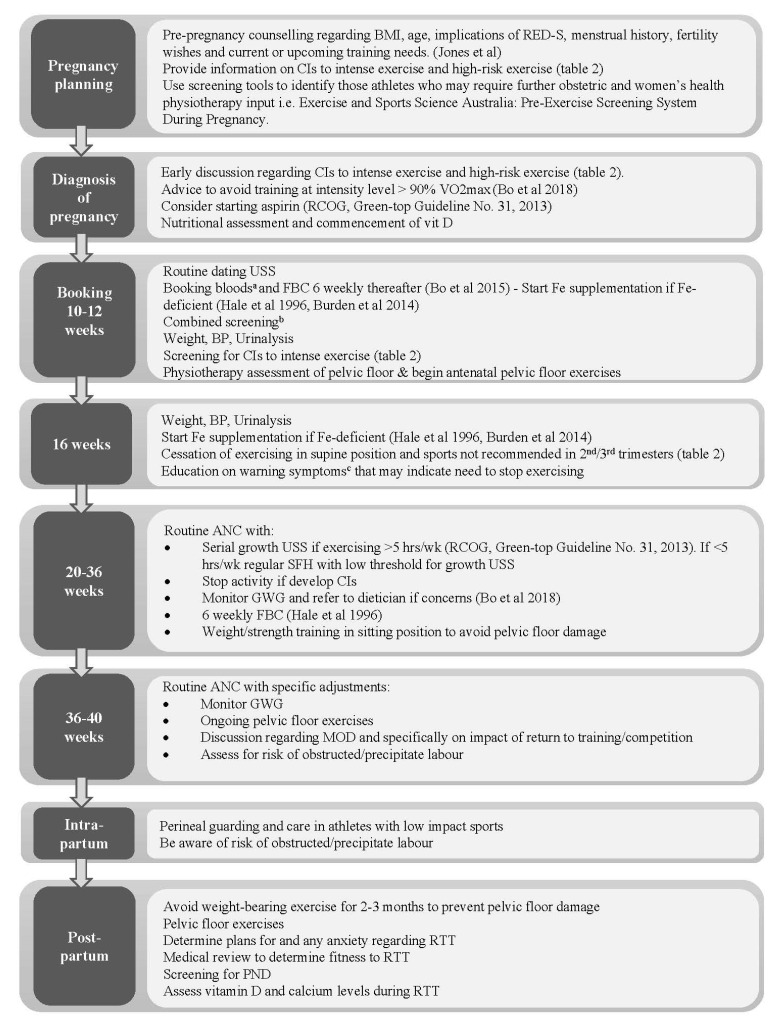
**Guidance for obstetricians managing athletes during the preconception, antenatal and postnatal periods:** ANC; antenatal care, BP; blood pressure CI; contraindication, FBC; full blood count, Fe; iron, GWG; gestational weight gain, hrs/wk; hours per week, MOD; mode of delivery; PND; postnatal depression, RED-S; relative energy deficiency in sport, RTT; return to training; USS; ultrasound scan, VO2 max; maximum oxygen consumption [11,13,65,66,83]. **^a^ Booking bloods;** FBC, Group and save, Syphilis, Hepatitis B, HIV, urea & electrolytes, glucose, iron studies, B12, folate **^b^ Combined screening;** Nuchal Translucency (NT)/Pregnancy Associated Plasma Protein A (PAPP-A)/hCG **^c^ Warning symptoms [42]**: Vaginal bleeding, regular painful contractions, leakage of amniotic fluid, dyspnoea before exercise, pre-syncopal symptoms or syncope, headache, chest pain, muscle weakness, calf pain/swelling.

The IOC previously published summary recommendations for elite athletes and recreational exercisers who exercise during pregnancy and postpartum [83]. Meah and colleagues called for a revision of previously recommended contraindications and provided overwhelming evidence for removing factors such as recurrent miscarriage from the list [39]. A summary of the suggested contraindications from both groups is provided in Table 2 [81]. These recommendations, along with recent guidelines [1,3,4,6], and the evidence provided herein, have been used to formulate guidance for obstetricians managing athletes preconceptionally and throughout the antenatal and postnatal periods, as summarised in Figure 1. In addition to routine obstetric care, the overriding aim is on the minimalisation of maternal and foetal risks associated with high-intensity training whilst supporting women to safely optimise athletic performance. The decision to return to exercise postpartum needs to be individualised, with consideration of various factors such as wound healing, anaemia, fatigue, breastfeeding, and any obstetric issues. Athletes returning to training after childbirth should seek advice from their women’s health physiotherapists to ensure they are medically fit, who should adopt a multidisciplinary, individualised approach.

## 9. Conclusions

In recent years our understanding of how exercise affects pregnancy has evolved concurrently with increased participation. However, data on elite athletes remain limited, which may be related to pregnancy remaining less common in this group owing to reproductive planning around careers. Overall, there is little evidence to suggest that elite-level sport leads to adverse pregnancy outcomes; whilst this may be due to the small study numbers in this cohort, data from larger studies looking at different levels of intensity of exercise do not suggest an increase in adverse outcomes with increasing intensity. Sportswomen should therefore be supported to continue their training, with adjustments to intensity and type of activity where appropriate, in addition to adequate supervision and monitoring from healthcare professionals. Whilst data remain limited, there does not appear to be a significant deterioration in sports performance at early gestations, which may be enhanced by pregnancy-associated physiological cardiovascular adaptations. However, as gestation advances, performance level decreases secondary to weight gain, anaemia and increased joint laxity.

## Figures and Tables

**Table 1:** **Physiological changes in pregnancy and implications for exercise** [11,14,15,19,20,21,22,23,24,25,26,27]. CO; cardiac output, E; oestrogen, Hb; haemoglobin, HR; heart rate, IVC; inferior vena cava, PVR; peripheral vascular resistance, SV; stroke volume, VO2max; maximal oxygen consumption.

Physiological Adaptation	1st Trimester	2nd Trimester	3rd Trimester	Implication for Exercise
**Cardiovascular**	Increase in CO by 20% Marked fall in PVR Increase in blood volume	Maximum CO (40% increase) at 20–28 week Fall in PVR by 25–30	Minimal fall in CO at term, with SV declining but raised HR persisting PVR increases from 32 weeks Blood volume increases to 50% of pre-pregnancy level by term In supine position, pressure of gravid uterus on IVC reduces venous return to the heart, with reduced SV and C	Compensation for moderate but not strenuous physical activity or endurance sports Misinterpretation of maximum HR Difficulty exercising in supine position Decline in oxygen carrying capacity of blood due to haemodilution. Improved performance postpartum
	**Throughout**: Hypervolaemia due to increased E, which activates renin-angiotensin system, as well as involvement of other hormones			
**Respiratory**	Reduced inspiratory reserve volume due to increased tidal volume		20–30% increase in VO2 max at term Diaphragmatic elevation causes decreased functional residual capacity Inspiratory reserve volume increases due to reduced functional residual capacity	Overall positive impact on exercise due to increase in VO2max At later gestation, subjective feeling of hypoxia at rest, which can improve with mild activity
	**Throughout**: Increase in minute ventilation (40–50%) and VO2max.			
**Haematological**		Increased plasma volume with steady fall in Hb throughout 2nd trimester	Plasma volume increased by 50% by 34 weeks Haemoconcentration towards ter	Altered perception of exertion
	**Throughout**: Physiological hypercoagulable state			
**Musculoskeletal**			Exaggerated lordosis of the lower back, forward flexion of the neck and downward movement of the shoulders Increased mobility and widening of sacroiliac joints and pubic symphysis. Increased joint laxity due to increased oestrogen and relaxin levels Gravid uterus can result in shift in centre of gravity	Discomfort or damage to joints Increased risk of injury or falls, but improved recovery following injury Lumbar lordosis causes change in centre of gravity and pelvis to tilt forwards; can cause injury, particularly in stop-and-go training
	**Throughout**: Increasing E concentrations potentially improves muscle and tendon strength Change in the micro-architectural pattern of bone in pregnancy but not overall bone mass Pregnancy-associated weight gain increases forces across joints			
**Metabolic/endocrine**	Total cortisol levels increase at the end of the 1st trimester Increased insulin secretion and increased insulin sensitivity in early pregnancy	Insulin resistance begins	Cortisol level 3x that of non-pregnant values at end of pregnancy Peak insulin resistance	Increased maternal nutritional requirements beyond the extra 300 kcal daily intake required to maintain the metabolic demands of pregnancy
	**Throughout**: 15% increase in metabolic rate State of hypercortisolism Insulin-resistant state to allow glucose availability for fetus.			

**Table 2:** **Contraindications to aerobic exercise during pregnancy [39,83]**: IOC; International Olympic Committee, FGR; intrauterine growth restriction, PTL; preterm labour, T1DM; type 1 diabetes mellitus.

	Pre-existing Medical Conditions	Pregnancy-Related Conditions
**Absolute** (conditions posing high risk to fetus)	Haemodynamically significant heart disease (acquired/congenital) Uncontrolled arryhthmia Severe respiratory disease Uncontrolled T1DM ^Ψ^ Poorly controlled hypertension ^Δ^ Severe anaemia ^Δ^	Placental abruption FGR in current pregnancy Vasa praevia Cervical insufficiency/cerclage Severe pre-eclampsia PTL during current pregnancy Severe anaemia ^Δ^ Persistent bleeding in 2nd/3rd trimesters ^Δ^ Multiple gestation with risk of PTL ^Δ^ Severe anaemia ^Δ^
**Relative** (conditions posing moderate risk to fetus)	Mild respiratory disorders Mild congenital or acquired heart disease Well-controlled T1DM Untreated thyroid disease Symptomatic, severe eating disorder Multiple nutrient deficiencies and/or chronic undernutrition Orthopaedic limitations ^Δ^ Poorly controlled seizure disorder	Mild pre-eclampsia Preterm prelabour rupture of membranes * Placenta praevia > 28 weeks’ gestation * Previous fetal growth restriction, miscarriage, PTL or preterm birth ^Δ^
**No longer considered** **contraindications** **according to Meah et al. [39].**	Chronic hypertension Recurrent miscarriage Epilepsy Anaemia Hx of spontaneous PTL Hx of previous FGR	Pregnancy-induced hypertension Short cervix Multiple pregnancy

* denotes considered to be absolute contraindication by IOC. ^Ψ^ denotes considered to be relative contraindication by IOC. ^Δ^ denotes only considered to be contraindication by IOC.
